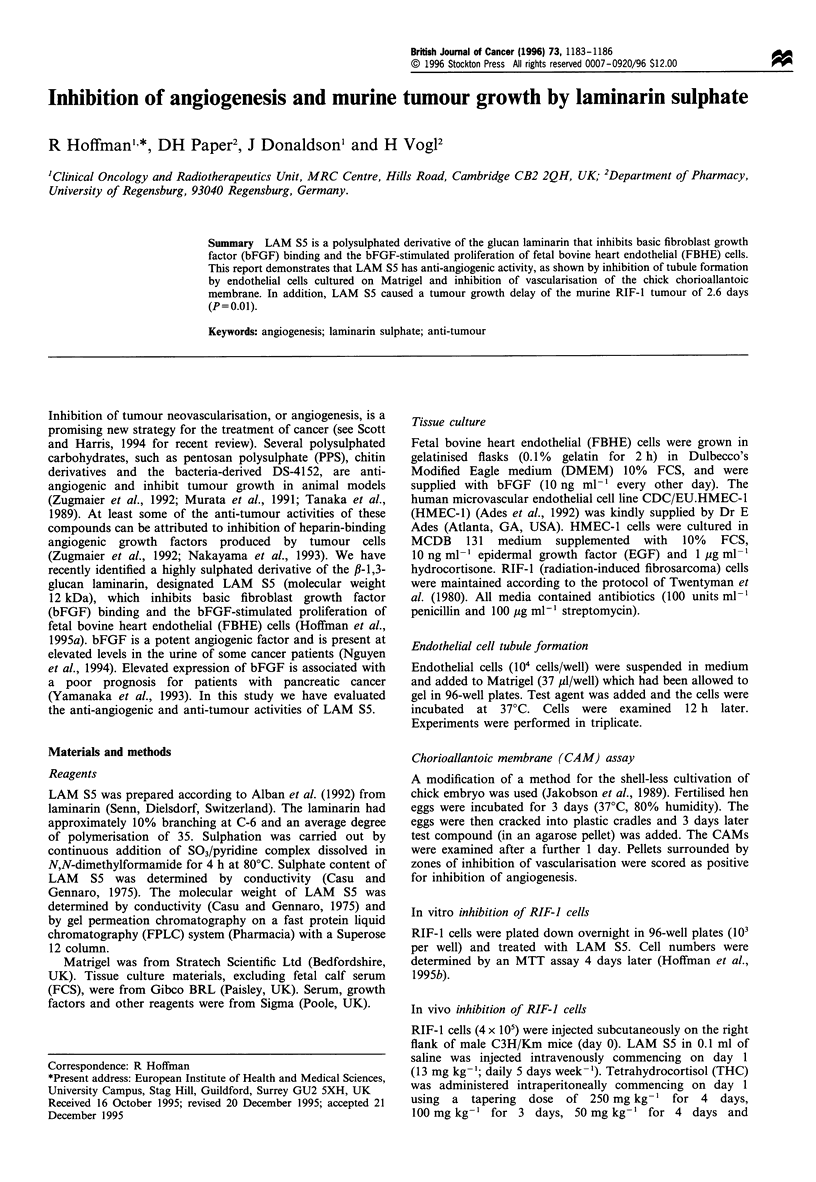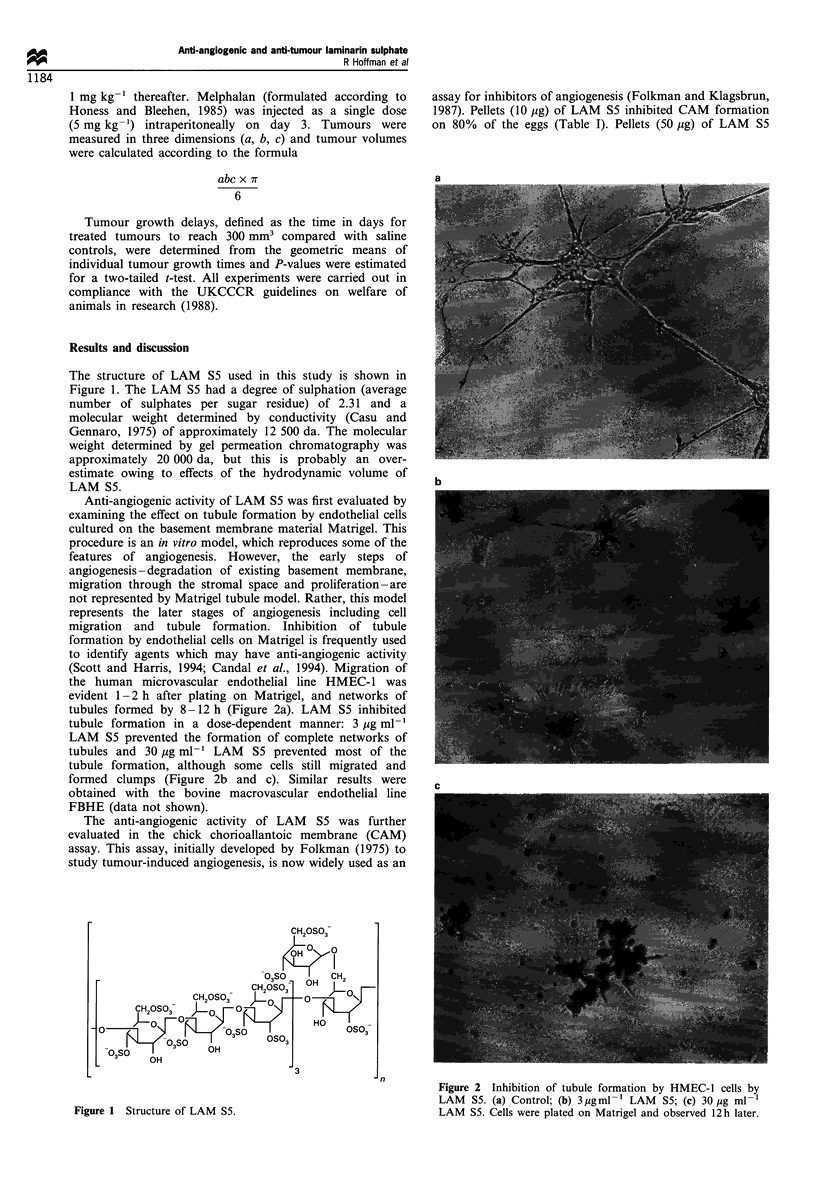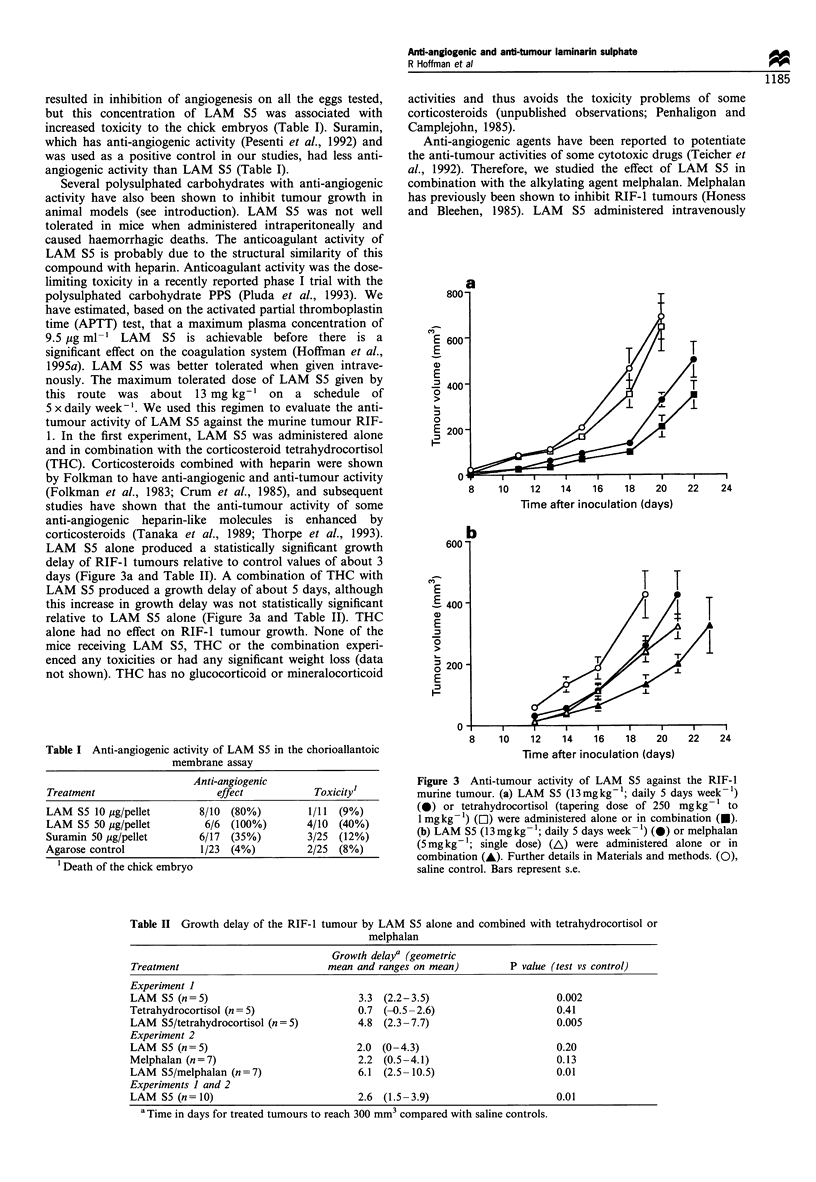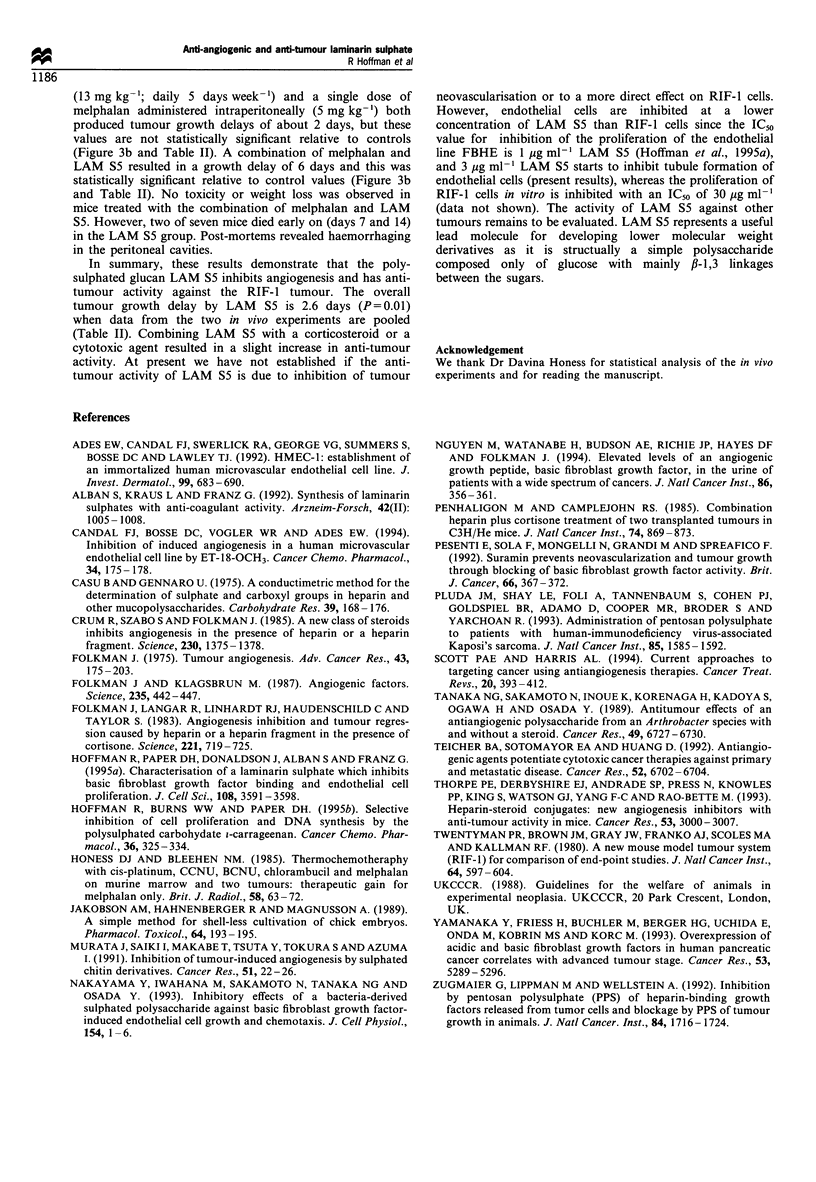# Inhibition of angiogenesis and murine tumour growth by laminarin sulphate.

**DOI:** 10.1038/bjc.1996.228

**Published:** 1996-05

**Authors:** R. Hoffman, D. H. Paper, J. Donaldson, H. Vogl

**Affiliations:** Clinical Oncology and Radiotherapeutics Unit, MRC Centre, Cambridge, UK.

## Abstract

**Images:**


British Journal of Cancer (1996) 73, 1183-1186
© 1996 Stockton Press All rights reserved 0007-0920/96 $12.00

### Inhibition of angiogenesis and murine tumour growth by laminarin sulphate

R Hoffman[1,*], DH Paper[2], J Donaldson[1] and H Vogl[2]

[1]*Clinical Oncology and Radiotherapeutics Unit, MRC Centre, Hills Road, Cambridge CB2 2QH, UK;* [2]*Department of Pharmacy, University of Regensburg, 93040 Regensburg, Germany.*

**Summary** LAM S5 is a polysulphated derivative of the glucan laminarin that inhibits basic fibroblast growth factor (bFGF) binding and the bFGF-stimulated proliferation of fetal bovine heart endothelial (FBHE) cells. This report demonstrates that LAM S5 has anti-angiogenic activity, as shown by inhibition of tubule formation by endothelial cells cultured on Matrigel and inhibition of vascularisation of the chick chorioallantoic membrane. In addition, LAM S5 caused a tumour growth delay of the murine RIF-1 tumour of 2.6 days ($P=0.01$).

**Keywords:** angiogenesis; laminarin sulphate; anti-tumour

Inhibition of tumour neovascularisation, or angiogenesis, is a promising new strategy for the treatment of cancer (see Scott and Harris, 1994 for recent review). Several polysulphated carbohydrates, such as pentosan polysulphate (PPS), chitin derivatives and the bacteria-derived DS-4152, are anti-angiogenic and inhibit tumour growth in animal models (Zugmaier *et al.*, 1992; Murata *et al.*, 1991; Tanaka *et al.*, 1989). At least some of the anti-tumour activities of these compounds can be attributed to inhibition of heparin-binding angiogenic growth factors produced by tumour cells (Zugmaier *et al.*, 1992; Nakayama *et al.*, 1993). We have recently identified a highly sulphated derivative of the β-1,3-glucan laminarin, designated LAM S5 (molecular weight 12 kDa), which inhibits basic fibroblast growth factor (bFGF) binding and the bFGF-stimulated proliferation of fetal bovine heart endothelial (FBHE) cells (Hoffman *et al.*, 1995*a*). bFGF is a potent angiogenic factor and is present at elevated levels in the urine of some cancer patients (Nguyen *et al.*, 1994). Elevated expression of bFGF is associated with a poor prognosis for patients with pancreatic cancer (Yamanaka *et al.*, 1993). In this study we have evaluated the anti-angiogenic and anti-tumour activities of LAM S5.

### Materials and methods

### *Reagents*

LAM S5 was prepared according to Alban *et al.* (1992) from laminarin (Senn, Dielsdorf, Switzerland). The laminarin had approximately 10% branching at C-6 and an average degree of polymerisation of 35. Sulphation was carried out by continuous addition of $SO_3$/pyridine complex dissolved in *N*,*N*-dimethylformamide for 4 h at 80°C. Sulphate content of LAM S5 was determined by conductivity (Casu and Gennaro, 1975). The molecular weight of LAM S5 was determined by conductivity (Casu and Gennaro, 1975) and by gel permeation chromatography on a fast protein liquid chromatography (FPLC) system (Pharmacia) with a Superose 12 column.

Matrigel was from Stratech Scientific Ltd (Bedfordshire, UK). Tissue culture materials, excluding fetal calf serum (FCS), were from Gibco BRL (Paisley, UK). Serum, growth factors and other reagents were from Sigma (Poole, UK).

Correspondence: R Hoffman
*Present address: European Institute of Health and Medical Sciences, University Campus, Stag Hill, Guildford, Surrey GU2 5XH, UK
Received 16 October 1995; revised 20 December 1995; accepted 21 December 1995

### *Tissue culture*

Fetal bovine heart endothelial (FBHE) cells were grown in gelatinised flasks (0.1% gelatin for 2 h) in Dulbecco's Modified Eagle medium (DMEM) 10% FCS, and were supplied with bFGF (10 ng $ml^{-1}$ every other day). The human microvascular endothelial cell line CDC/EU.HMEC-1 (HMEC-1) (Ades *et al.*, 1992) was kindly supplied by Dr E Ades (Atlanta, GA, USA). HMEC-1 cells were cultured in MCDB 131 medium supplemented with 10% FCS, 10 ng $ml^{-1}$ epidermal growth factor (EGF) and 1 μg $ml^{-1}$ hydrocortisone. RIF-1 (radiation-induced fibrosarcoma) cells were maintained according to the protocol of Twentyman *et al.* (1980). All media contained antibiotics (100 units $ml^{-1}$ penicillin and 100 μg $ml^{-1}$ streptomycin).

### *Endothelial cell tubule formation*

Endothelial cells ($10^4$ cells/well) were suspended in medium and added to Matrigel (37 μl/well) which had been allowed to gel in 96-well plates. Test agent was added and the cells were incubated at 37°C. Cells were examined 12 h later. Experiments were performed in triplicate.

### *Chorioallantoic membrane (CAM) assay*

A modification of a method for the shell-less cultivation of chick embryo was used (Jakobson *et al.*, 1989). Fertilised hen eggs were incubated for 3 days (37°C, 80% humidity). The eggs were then cracked into plastic cradles and 3 days later test compound (in an agarose pellet) was added. The CAMs were examined after a further 1 day. Pellets surrounded by zones of inhibition of vascularisation were scored as positive for inhibition of angiogenesis.

### In vitro *inhibition of RIF-1 cells*

RIF-1 cells were plated down overnight in 96-well plates ($10^3$ per well) and treated with LAM S5. Cell numbers were determined by an MTT assay 4 days later (Hoffman *et al.*, 1995*b*).

### In vivo *inhibition of RIF-1 cells*

RIF-1 cells ($4 \times 10^5$) were injected subcutaneously on the right flank of male C3H/Km mice (day 0). LAM S5 in 0.1 ml of saline was injected intravenously commencing on day 1 (13 mg $kg^{-1}$; daily 5 days $week^{-1}$). Tetrahydrocortisol (THC) was administered intraperitoneally commencing on day 1 using a tapering dose of 250 mg $kg^{-1}$ for 4 days, 100 mg $kg^{-1}$ for 3 days, 50 mg $kg^{-1}$ for 4 days and

1 mg kg$^{-1}$ thereafter. Melphalan (formulated according to Honess and Bleehen, 1985) was injected as a single dose (5 mg kg$^{-1}$) intraperitoneally on day 3. Tumours were measured in three dimensions (*a*, *b*, *c*) and tumour volumes were calculated according to the formula

$$\frac{abc \times \pi}{6}$$

Tumour growth delays, defined as the time in days for treated tumours to reach 300 mm$^3$ compared with saline controls, were determined from the geometric means of individual tumour growth times and *P*-values were estimated for a two-tailed *t*-test. All experiments were carried out in compliance with the UKCCCR guidelines on welfare of animals in research (1988).

### Results and discussion

The structure of LAM S5 used in this study is shown in Figure 1. The LAM S5 had a degree of sulphation (average number of sulphates per sugar residue) of 2.31 and a molecular weight determined by conductivity (Casu and Gennaro, 1975) of approximately 12 500 da. The molecular weight determined by gel permeation chromatography was approximately 20 000 da, but this is probably an overestimate owing to effects of the hydrodynamic volume of LAM S5.

Anti-angiogenic activity of LAM S5 was first evaluated by examining the effect on tubule formation by endothelial cells cultured on the basement membrane material Matrigel. This procedure is an *in vitro* model, which reproduces some of the features of angiogenesis. However, the early steps of angiogenesis – degradation of existing basement membrane, migration through the stromal space and proliferation – are not represented by Matrigel tubule model. Rather, this model represents the later stages of angiogenesis including cell migration and tubule formation. Inhibition of tubule formation by endothelial cells on Matrigel is frequently used to identify agents which may have anti-angiogenic activity (Scott and Harris, 1994; Candal *et al.*, 1994). Migration of the human microvascular endothelial line HMEC-1 was evident 1 – 2 h after plating on Matrigel, and networks of tubules formed by 8 – 12 h (Figure 2a). LAM S5 inhibited tubule formation in a dose-dependent manner: 3 $\mu$g ml$^{-1}$ LAM S5 prevented the formation of complete networks of tubules and 30 $\mu$g ml$^{-1}$ LAM S5 prevented most of the tubule formation, although some cells still migrated and formed clumps (Figure 2b and c). Similar results were obtained with the bovine macrovascular endothelial line FBHE (data not shown).

The anti-angiogenic activity of LAM S5 was further evaluated in the chick chorioallantoic membrane (CAM) assay. This assay, initially developed by Folkman (1975) to study tumour-induced angiogenesis, is now widely used as an assay for inhibitors of angiogenesis (Folkman and Klagsbrun, 1987). Pellets (10 $\mu$g) of LAM S5 inhibited CAM formation on 80% of the eggs (Table I). Pellets (50 $\mu$g) of LAM S5

**Figure 1** Structure of LAM S5.

**Figure 2** Inhibition of tubule formation by HMEC-1 cells by LAM S5. (**a**) Control; (**b**) 3 $\mu$g ml$^{-1}$ LAM S5; (**c**) 30 $\mu$g ml$^{-1}$ LAM S5. Cells were plated on Matrigel and observed 12 h later.

resulted in inhibition of angiogenesis on all the eggs tested, but this concentration of LAM S5 was associated with increased toxicity to the chick embryos (Table I). Suramin, which has anti-angiogenic activity (Pesenti *et al.*, 1992) and was used as a positive control in our studies, had less anti-angiogenic activity than LAM S5 (Table I).

Several polysulphated carbohydrates with anti-angiogenic activity have also been shown to inhibit tumour growth in animal models (see introduction). LAM S5 was not well tolerated in mice when administered intraperitoneally and caused haemorrhagic deaths. The anticoagulant activity of LAM S5 is probably due to the structural similarity of this compound with heparin. Anticoagulant activity was the dose-limiting toxicity in a recently reported phase I trial with the polysulphated carbohydrate PPS (Pluda *et al.*, 1993). We have estimated, based on the activated partial thromboplastin time (APTT) test, that a maximum plasma concentration of 9.5 $\mu g\ ml^{-1}$ LAM S5 is achievable before there is a significant effect on the coagulation system (Hoffman *et al.*, 1995*a*). LAM S5 was better tolerated when given intravenously. The maximum tolerated dose of LAM S5 given by this route was about 13 $mg\ kg^{-1}$ on a schedule of $5 \times$ daily $week^{-1}$. We used this regimen to evaluate the anti-tumour activity of LAM S5 against the murine tumour RIF-1. In the first experiment, LAM S5 was administered alone and in combination with the corticosteroid tetrahydrocortisol (THC). Corticosteroids combined with heparin were shown by Folkman to have anti-angiogenic and anti-tumour activity (Folkman *et al.*, 1983; Crum *et al.*, 1985), and subsequent studies have shown that the anti-tumour activity of some anti-angiogenic heparin-like molecules is enhanced by corticosteroids (Tanaka *et al.*, 1989; Thorpe *et al.*, 1993). LAM S5 alone produced a statistically significant growth delay of RIF-1 tumours relative to control values of about 3 days (Figure 3a and Table II). A combination of THC with LAM S5 produced a growth delay of about 5 days, although this increase in growth delay was not statistically significant relative to LAM S5 alone (Figure 3a and Table II). THC alone had no effect on RIF-1 tumour growth. None of the mice receiving LAM S5, THC or the combination experienced any toxicities or had any significant weight loss (data not shown). THC has no glucocorticoid or mineralocorticoid activities and thus avoids the toxicity problems of some corticosteroids (unpublished observations; Penhaligon and Camplejohn, 1985).

Anti-angiogenic agents have been reported to potentiate the anti-tumour activities of some cytotoxic drugs (Teicher *et al.*, 1992). Therefore, we studied the effect of LAM S5 in combination with the alkylating agent melphalan. Melphalan has previously been shown to inhibit RIF-1 tumours (Honess and Bleehen, 1985). LAM S5 administered intravenously

**Table I** Anti-angiogenic activity of LAM S5 in the chorioallantoic membrane assay

| *Treatment* | *Anti-angiogenic effect* | *Toxicity*[1] |
|---|---|---|
| LAM S5 10 μg/pellet | 8/10 (80%) | 1/11 (9%) |
| LAM S5 50 μg/pellet | 6/6 (100%) | 4/10 (40%) |
| Suramin 50 μg/pellet | 6/17 (35%) | 3/25 (12%) |
| Agarose control | 1/23 (4%) | 2/25 (8%) |

[1] Death of the chick embryo

**Figure 3** Anti-tumour activity of LAM S5 against the RIF-1 murine tumour. (**a**) LAM S5 ($13\ mg\ kg^{-1}$; daily 5 days $week^{-1}$) (●) or tetrahydrocortisol (tapering dose of 250 $mg\ kg^{-1}$ to $1\ mg\ kg^{-1}$) (□) were administered alone or in combination (■). (**b**) LAM S5 ($13\ mg\ kg^{-1}$; daily 5 days $week^{-1}$) (●) or melphalan ($5\ mg\ kg^{-1}$; single dose) (△) were administered alone or in combination (▲). Further details in Materials and methods. (○), saline control. Bars represent s.e.

**Table II** Growth delay of the RIF-1 tumour by LAM S5 alone and combined with tetrahydrocortisol or melphalan

| *Treatment* | *Growth delay*[a] *(geometric mean and ranges on mean)* | P *value (test vs control)* |
|---|---|---|
| *Experiment 1* | | |
| LAM S5 (*n* = 5) | 3.3 (2.2–3.5) | 0.002 |
| Tetrahydrocortisol (*n* = 5) | 0.7 (–0.5–2.6) | 0.41 |
| LAM S5/tetrahydrocortisol (*n* = 5) | 4.8 (2.3–7.7) | 0.005 |
| *Experiment 2* | | |
| LAM S5 (*n* = 5) | 2.0 (0–4.3) | 0.20 |
| Melphalan (*n* = 7) | 2.2 (0.5–4.1) | 0.13 |
| LAM S5/melphalan (*n* = 7) | 6.1 (2.5–10.5) | 0.01 |
| *Experiments 1 and 2* | | |
| LAM S5 (*n* = 10) | 2.6 (1.5–3.9) | 0.01 |

[a] Time in days for treated tumours to reach 300 $mm^3$ compared with saline controls.

(13 mg $kg^{-1}$; daily 5 days $week^{-1}$) and a single dose of melphalan administered intraperitoneally (5 mg $kg^{-1}$) both produced tumour growth delays of about 2 days, but these values are not statistically significant relative to controls (Figure 3b and Table II). A combination of melphalan and LAM S5 resulted in a growth delay of 6 days and this was statistically significant relative to control values (Figure 3b and Table II). No toxicity or weight loss was observed in mice treated with the combination of melphalan and LAM S5. However, two of seven mice died early on (days 7 and 14) in the LAM S5 group. Post-mortems revealed haemorrhaging in the peritoneal cavities.

In summary, these results demonstrate that the polysulphated glucan LAM S5 inhibits angiogenesis and has anti-tumour activity against the RIF-1 tumour. The overall tumour growth delay by LAM S5 is 2.6 days ($P=0.01$) when data from the two *in vivo* experiments are pooled (Table II). Combining LAM S5 with a corticosteroid or a cytotoxic agent resulted in a slight increase in anti-tumour activity. At present we have not established if the anti-tumour activity of LAM S5 is due to inhibition of tumour neovascularisation or to a more direct effect on RIF-1 cells. However, endothelial cells are inhibited at a lower concentration of LAM S5 than RIF-1 cells since the $IC_{50}$ value for inhibition of the proliferation of the endothelial line FBHE is 1 $\mu g\ ml^{-1}$ LAM S5 (Hoffman *et al.*, 1995*a*), and 3 $\mu g\ ml^{-1}$ LAM S5 starts to inhibit tubule formation of endothelial cells (present results), whereas the proliferation of RIF-1 cells *in vitro* is inhibited with an $IC_{50}$ of 30 $\mu g\ ml^{-1}$ (data not shown). The activity of LAM S5 against other tumours remains to be evaluated. LAM S5 represents a useful lead molecule for developing lower molecular weight derivatives as it is structually a simple polysaccharide composed only of glucose with mainly $\beta$-1,3 linkages between the sugars.

**Acknowledgement**
We thank Dr Davina Honess for statistical analysis of the *in vivo* experiments and for reading the manuscript.

### References

ADES EW, CANDAL FJ, SWERLICK RA, GEORGE VG, SUMMERS S, BOSSE DC AND LAWLEY TJ. (1992). HMEC-1: establishment of an immortalized human microvascular endothelial cell line. *J. Invest. Dermatol.*, **99**, 683–690.

ALBAN S, KRAUS L AND FRANZ G. (1992). Synthesis of laminarin sulphates with anti-coagulant activity. *Arzneim-Forsch*, **42**(II): 1005–1008.

CANDAL FJ, BOSSE DC, VOGLER WR AND ADES EW. (1994). Inhibition of induced angiogenesis in a human microvascular endothelial cell line by ET-18-$OCH_3$. *Cancer Chemo. Pharmacol.*, **34**, 175–178.

CASU B AND GENNARO U. (1975). A conductimetric method for the determination of sulphate and carboxyl groups in heparin and other mucopolysaccharides. *Carbohydrate Res.* **39**, 168–176.

CRUM R, SZABO S AND FOLKMAN J. (1985). A new class of steroids inhibits angiogenesis in the presence of heparin or a heparin fragment. *Science*, **230**, 1375–1378.

FOLKMAN J. (1975). Tumour angiogenesis. *Adv. Cancer Res.*, **43**, 175–203.

FOLKMAN J AND KLAGSBRUN M. (1987). Angiogenic factors. *Science*, **235**, 442–447.

FOLKMAN J, LANGAR R, LINHARDT RJ, HAUDENSCHILD C AND TAYLOR S. (1983). Angiogenesis inhibition and tumour regression caused by heparin or a heparin fragment in the presence of cortisone. *Science*, **221**, 719–725.

HOFFMAN R, PAPER DH, DONALDSON J, ALBAN S AND FRANZ G. (1995*a*). Characterisation of a laminarin sulphate which inhibits basic fibroblast growth factor binding and endothelial cell proliferation. *J. Cell Sci.*, **108**, 3591–3598.

HOFFMAN R, BURNS WW AND PAPER DH. (1995*b*). Selective inhibition of cell proliferation and DNA synthesis by the polysulphated carbohydate ι-carrageenan. *Cancer Chemo. Pharmacol.*, **36**, 325–334.

HONESS DJ AND BLEEHEN NM. (1985). Thermochemotheraphy with cis-platinum, CCNU, BCNU, chlorambucil and melphalan on murine marrow and two tumours: therapeutic gain for melphalan only. *Brit. J. Radiol.*, **58**, 63–72.

JAKOBSON AM, HAHNENBERGER R AND MAGNUSSON A. (1989). A simple method for shell-less cultivation of chick embryos. *Pharmacol. Toxicol.*, **64**, 193–195.

MURATA J, SAIKI I, MAKABE T, TSUTA Y, TOKURA S AND AZUMA I. (1991). Inhibition of tumour-induced angiogenesis by sulphated chitin derivatives. *Cancer Res.*, **51**, 22–26.

NAKAYAMA Y, IWAHANA M, SAKAMOTO N, TANAKA NG AND OSADA Y. (1993). Inhibitory effects of a bacteria-derived sulphated polysaccharide against basic fibroblast growth factor-induced endothelial cell growth and chemotaxis. *J. Cell Physiol.*, **154**, 1–6.

NGUYEN M, WATANABE H, BUDSON AE, RICHIE JP, HAYES DF AND FOLKMAN J. (1994). Elevated levels of an angiogenic growth peptide, basic fibroblast growth factor, in the urine of patients with a wide spectrum of cancers. *J. Natl Cancer Inst.*, **86**, 356–361.

PENHALIGON M AND CAMPLEJOHN RS. (1985). Combination heparin plus cortisone treatment of two transplanted tumours in C3H/He mice. *J. Natl Cancer Inst.*, **74**, 869–873.

PESENTI E, SOLA F, MONGELLI N, GRANDI M AND SPREAFICO F. (1992). Suramin prevents neovascularization and tumour growth through blocking of basic fibroblast growth factor activity. *Brit. J. Cancer*, **66**, 367–372.

PLUDA JM, SHAY LE, FOLI A, TANNENBAUM S, COHEN PJ, GOLDSPIEL BR, ADAMO D, COOPER MR, BRODER S AND YARCHOAN R. (1993). Administration of pentosan polysulphate to patients with human-immunodeficiency virus-associated Kaposi's sarcoma. *J. Natl Cancer Inst.*, **85**, 1585–1592.

SCOTT PAE AND HARRIS AL. (1994). Current approaches to targeting cancer using antiangiogenesis therapies. *Cancer Treat. Revs.*, **20**, 393–412.

TANAKA NG, SAKAMOTO N, INOUE K, KORENAGA H, KADOYA S, OGAWA H AND OSADA Y. (1989). Antitumour effects of an antiangiogenic polysaccharide from an *Arthrobacter* species with and without a steroid. *Cancer Res.*, **49**, 6727–6730.

TEICHER BA, SOTOMAYOR EA AND HUANG D. (1992). Antiangiogenic agents potentiate cytotoxic cancer therapies against primary and metastatic disease. *Cancer Res.*, **52**, 6702–6704.

THORPE PE, DERBYSHIRE EJ, ANDRADE SP, PRESS N, KNOWLES PP, KING S, WATSON GJ, YANG F-C AND RAO-BETTE M. (1993). Heparin-steroid conjugates: new angiogenesis inhibitors with anti-tumour activity in mice. *Cancer Res.*, **53**, 3000–3007.

TWENTYMAN PR, BROWN JM, GRAY JW, FRANKO AJ, SCOLES MA AND KALLMAN RF. (1980). A new mouse model tumour system (RIF-1) for comparison of end-point studies. *J. Natl Cancer Inst.*, **64**, 597–604.

UKCCCR. (1988). Guidelines for the welfare of animals in experimental neoplasia. UKCCCR, 20 Park Crescent, London, UK.

YAMANAKA Y, FRIESS H, BUCHLER M, BERGER HG, UCHIDA E, ONDA M, KOBRIN MS AND KORC M. (1993). Overexpression of acidic and basic fibroblast growth factors in human pancreatic cancer correlates with advanced tumour stage. *Cancer Res.*, **53**, 5289–5296.

ZUGMAIER G, LIPPMAN M AND WELLSTEIN A. (1992). Inhibition by pentosan polysulphate (PPS) of heparin-binding growth factors released from tumor cells and blockage by PPS of tumour growth in animals. *J. Natl Cancer. Inst.*, **84**, 1716–1724.